# Rebuilding hippocampus neural circuit with hADSC-derived neuron cells for treating ischemic stroke

**DOI:** 10.1186/s13578-022-00774-x

**Published:** 2022-04-04

**Authors:** Jian Wang, Rui Hao, Tianfang Jiang, Xuanxuan Guo, Fei Zhou, Limei Cao, Fengjuan Gao, Guangming Wang, Juan Wang, Ke Ning, Chunlong Zhong, Xu Chen, Ying Huang, Jun Xu, Shane Gao

**Affiliations:** 1 Department of Neurosurgery, Shanghai East Hospital, Tongji University School of Medicine, Shanghai, 200120 China; 2 Center of Translational Medicine, Tongji Hospital, Tongji University School of Medicine, Shanghai, 200092 China; 3 Department of Neurology, Shanghai Eighth People’s Hospital Affiliated to Jiangsu University, Shanghai, 200233 China; 4 Department of Neurology, Third Affiliated Hospital of Navy Military Medical University, Shanghai, 200438 China; 5 Zhoupu Hospital, Affiliated to Shanghai University of Medicine & Health Sciences, Shanghai, 201318 China; 6 Department of Biotechnology and Molecular, Binzhou Medical College, Yantai, 264003 Shandong People’s Republic of China; 7 Department of Neuroscience, Sheffield Institute for Translational Neuroscience (SITraN), University of Sheffield, 385A Glossop Road, Sheffield, S10 2HQ UK; 8 Department of Anesthesia, Zhongshan Hospital, Fudan University, Shanghai,, 200032, China

**Keywords:** Adipose-derived stem cells (ADSCs), hADSC-derived neuron-like cells (hADSC-NCs), Middle cerebral artery occlusion (MCAO), National Institute of Health Stroke Scale (NIHSS), Rogers scale system

## Abstract

**Background:**

Human adipose-derived stem cells (hADSCs) have been demonstrated to be a promising autologous stem cell source for treating various neuronal diseases. Our study indicated that hADSCs could be induced into neuron-like cells in a stepwise manner that are characterized by the positive expression of MAP2, SYNAPSIN 1/2, NF-200, and vGLUT and electrophysiological activity. We first primed hADSCs into neuron-like cells (hADSC-NCs) and then intracerebrally transplanted them into MCAO reperfusion mice to further explore their in vivo survival, migration, integration, fate commitment and involvement in neural circuit rebuilding.

**Results:**

The hADSC-NCs survived well and transformed into MAP2-positive, Iba1- or GFAP-negative cells in vivo while maintaining some proliferative ability, indicated by positive Ki67 staining after 4 weeks. hADSC-NCs could migrate to multiple brain regions, including the cortex, hippocampus, striatum, and hypothalamus, and further differentiate into mature neurons, as confirmed by action potential elicitation and postsynaptic currents. With the aid of a cell suicide system, hADSC-NCs were proven to have functionally integrated into the hippocampal memory circuit, where they contributed to spatial learning and memory rescue, as indicated by LTP improvement and subsequent GCV-induced relapse. In addition to infarction size shrinkage and movement improvement, MCAO-reperfused mice showed bidirectional immune modulation, including inhibition of the local proinflammatory factors IL-1α, IL-1β, IL-2, MIP-1β and promotion proinflammatory IP-10, MCP-1, and enhancement of the anti-inflammatory factors IL-15.

**Conclusion:**

Overall, hADSC-NCs used as an intermediate autologous cell source for treating stroke can rebuild hippocampus neuronal circuits through cell replacement.

**Supplementary Information:**

The online version contains supplementary material available at 10.1186/s13578-022-00774-x.

## Introduction

Stroke ranks among the most devastating disease with high morbidity and mortality. Most stroke survivors suffer severe irreversible deficits, including motor, sense and cognition. Brain damage due to the disruption of the blood supply results from series of complex and cascading events, including glial cell over-reaction, blood–brain barrier (BBB) rupture, mitochondrial dysfunction, chronic inflammation, overproduction of free radicals, excitation toxicities, neuronal axonal and synaptic loss, demyelination, neural circuitry disruption and eventual behavioral deficits, disabilities or death. Currently, few treatments other than i.v. Recombinant Tissue Plasminogen Activator (rtPA) thrombolysis and antihypertensive therapy have been shown to be effective in clinical practice, and these treatments have a narrow therapeutic time window [31, 69]. Although other candidate drugs targeting neuroprotection, reduction of thrombosis and inflammation, such as erythropoietin (EPO) [17], NMDA receptor antagonists [46], GABA receptor agonists [45], thrombin inhibitors [2], and intracellular adhesion molecular 1 (ICAM-1) inhibitors [54], have demonstrated some therapeutic potential in preclinical studies, their clinical value is yet to be proven. Clinical cell therapies have shown some neurorestorative effects for patients with stroke, such as, olfactory ensheathing cell[24, 59] and other cells [27].

Human adipose-derived stem cells (hADSCs) possess many advantages as mesenchymal stem cells, such as the feasibility of autologous transplantation, easy and minimally invasive collection methods, enrichment in adipose tissue, and secretion ability, multidifferentiation potential and immune modulation capacities [37, 66, 73]. Recent studies have proven that hADSCs are a promising stem cell source for treating traumatic neural diseases such as stroke [22, 72] and various neural degenerative diseases including Alzheimer’s disease (AD) [36, 65], Parkinson’s disease (PD) [8, 10] and amyotrophic lateral sclerosis (ALS) [9, 38]. One of the most attractive features of hADSCs as a candidate cell source for treating neuronal diseases may be their capability for neuronal lineage differentiation, which makes in vivo cell replacement possible [20, 56, 68]. Previous publications reported the feasibility of transdifferentiating hADSCs into neuronal lineage cells in vitro [1, 21, 48, 49, 67]. Priming hADSCs to differentiate into neuronal lineage cells before transplantation may result in much greater chance of cell replacement in vivo [7, 8]. However, how these procedures affect the fate of hADSCs in vivo when interacting with the microenvironment has yet to be further explored.

Recent preclinical data on using hADSCs to treat stroke animal models demonstrated some positive therapeutic effects but inadequate in explaining the underlying mechanism [55]. Some data were collected from the intravenous injection of hADSCs, which showed promising for behavioral amelioration, but the transplanted hADSCs could not be traced to further reveal the real mechanism. Chen et al. [5] transplanted allogeneic ADSCs into the lateral ventricle of the rat brain after MCAO and observed that the ADSCs could survive in the brain parenchyma and express characteristic markers of neurons and glial cells [microtubule-associated protein 2 (MAP2 and glial fibrillary acidic protein (GFAP, respectively]. In addition, Wang et al. [58] reported that transplanted ADSCs expressed endothelial markers [von Willebrand factor (vWF) and endothelial barrier antigen] but not neuronal or glia markers. Meanwhile, other research groups reported that a low number of donor cells could be found in the lesion area or within the whole brain, suggesting that ADSCs are subject to apoptotic death or autophagia in vivo following their administration [33, 44]. However, preclinical data have shed lights on the clinical translation of hADSCs in later clinical trial studies [15]. Previous clinical publications have also demonstrated the safety and promising therapeutic effects of intravenous, intracerebral and intrathecal delivery of ADSCs [13, 16].

Evidence from recent animal studies hinted that the beneficial effects of stem cell therapy are partially due to mechanisms of such as paracrine [6, 32, 39, 40], immune modulation, anti-apoptosis and neurotrophic support mechanisms [11, 19, 70, 71]. Rat ADSCs have been shown to reduce the levels of IL-18, Toll-like receptor (TLR)-4, and plasminogen activator inhibitor-1 (PAhI-1) but to increase Bcl-2 and IL-8 levels in a rat model of acute ischemic stroke [41]. The in vivo data are consistent with the results of in vitro inflammation mimicking studies, revealing that ADSCs interfere with the immune system by suppressing the proliferation of peripheral blood mononuclear cells and inhibiting the differentiation of monocyte-derived immature dendritic cells [47, 52, 62]. Moreover, high levels of immune modulatory cytokines, such as IL-6 and transforming growth factor-1 (TGF-1), could be detected in in vitro cultures [30, 34]. These observations are highly relevant to functional recovery and should be analyzed in detail in future research. However, little is known about the exact immune-modulatory effects of ADSC administration in vivo after stroke [35]. ADSCs also have the capacity to secrete a wide spectrum of trophic factors, including brain-derived neurotrophic factor (BDNF), insulin-like growth factor-1 (IGF-1), vascular endothelial growth factor (VEGF), and hepatocyte growth factor (HGF), which may contribute to stroke symptom improvement in animal models by preventing neuronal apoptosis, enhancing the intrinsic repair response and modulating inflammation [23, 44, 50, 71].

Cell replacement in vivo is always highly anticipated for long-term regeneration in neuron degenerative diseases. The best way to utilize hADSCs in vitro to properly introduce them into the injury site in the brain of a mouse subjected to stroke and allow them to functionally integrate into the broken neural circuit is a challenge. In this study, we developed a strategy to expose hADSCs in a neuronal differentiation cocktail for short time in vitro and to transplant these primed hADSCs stereotactically into the injury site in the brain of a mouse subjected to MCAO reperfusion ischemic stroke. We hypothesized that manipulation of these cells in advance can turn on their neuronal switch in vivo while still maintaining their original immune-modulatory and neurotrophic capacities. Thus, we might develop a better therapeutic solution for stroke.

## Results

### hADSC characterization and gradual differentiation into neuron-like cells with electrophysiological activities in vitro

**Fig. 1:**
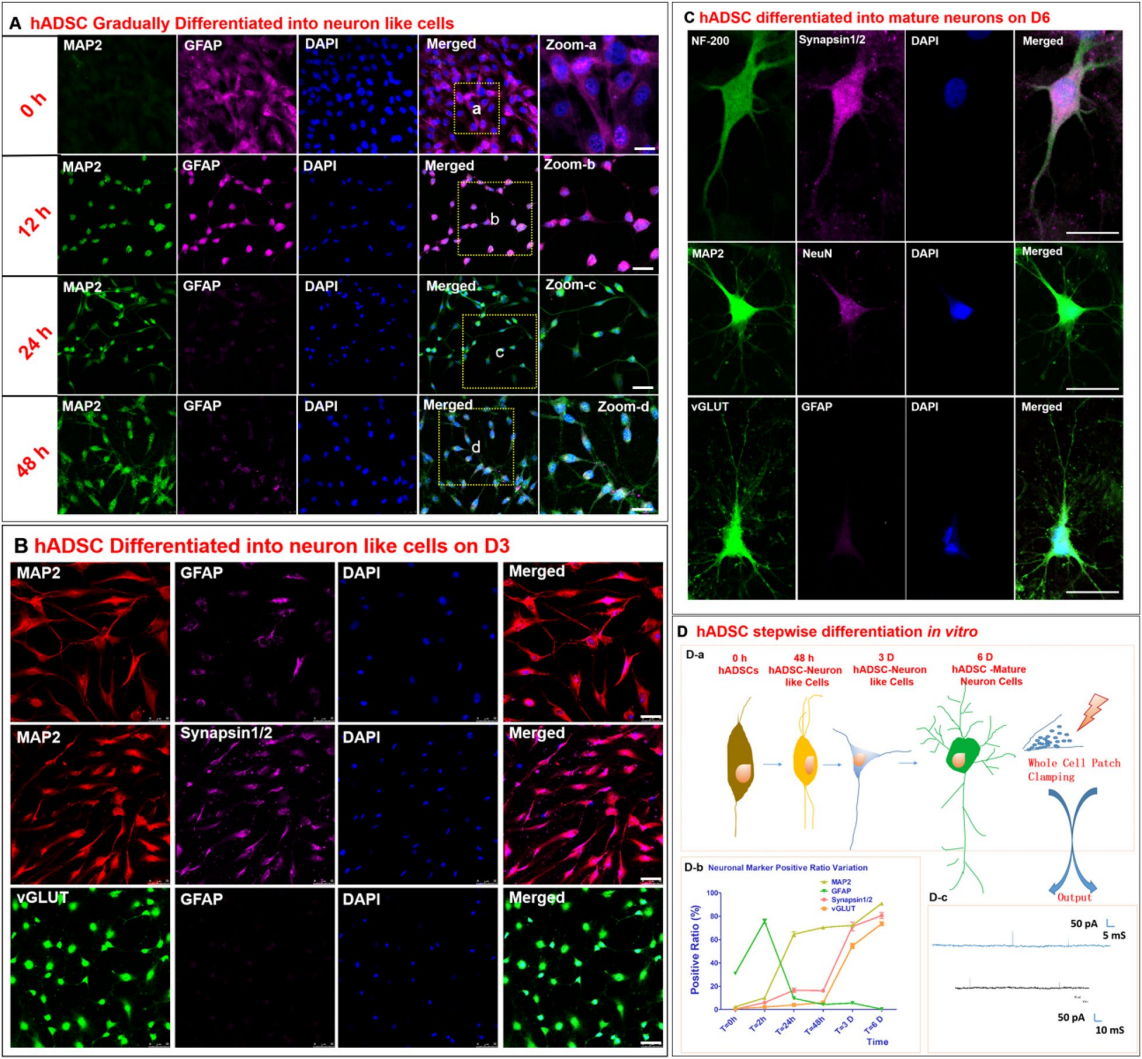
hADSCs are gradually induced to differentiate into neuron-like cells characterized by neuronal marker staining and action potentials. **A** Shows the variance in morphology and neuronal marker expression during stepwise induction from 0 to 48 h. **B** Shows that some functional neuronal markers could be detected and were distributed within the cell soma on day 3 (D3). **C** shows that hADSCs could be induced to differentiate into relatively mature neurons with complex neurites and neurotransmitters on Day 6 (D6). **D** Shows the cell morphological variance diagram (**D**-**a**), the change in the positive expression ratio over time for various neuronal markers (**D**-**b**) and the action potentials measured by whole-cell patch clamping on D6. n = 5, Scale bar = 50 μm in A-B, Scale bar = 25 μm in C

Our previous studies have stably set up hADSC isolation, characterization protocols [20, 72]. Previously, a optimized protocol to induce hADSCs into NCs was developed in our lab to analyze their developmental and electrophysiological activity variation [20]. Our studies showed that hADSCs could be gradually induced to differentiate into NCs, which showed the appropriate morphology as well as increased MAP2 and decreased GFAP expression within 48 h (Fig. 1A). On day 3, the NCs began to express the synaptic markers synapsin 1/2 and vGLUT (Fig. 1B). On day 6, the NCs became more mature with complex neurites and high expression of vGLUT and Synapsin 1/2 (Fig. 1C). The cell morphology and markers were determined and are summarized in Fig. 1D. On day 6, whole-cell patch clamp recordings were performed on these hADSC-NCs, and action potentials were detectible, suggesting that the NCs began to exhibit electrophysiological activities in vitro in addition to their expression of neuronal markers.

### hADSC-NC transplantation leads to dramatic behavioral improvement in MCAO reperfusion mice

**Fig. 2:**
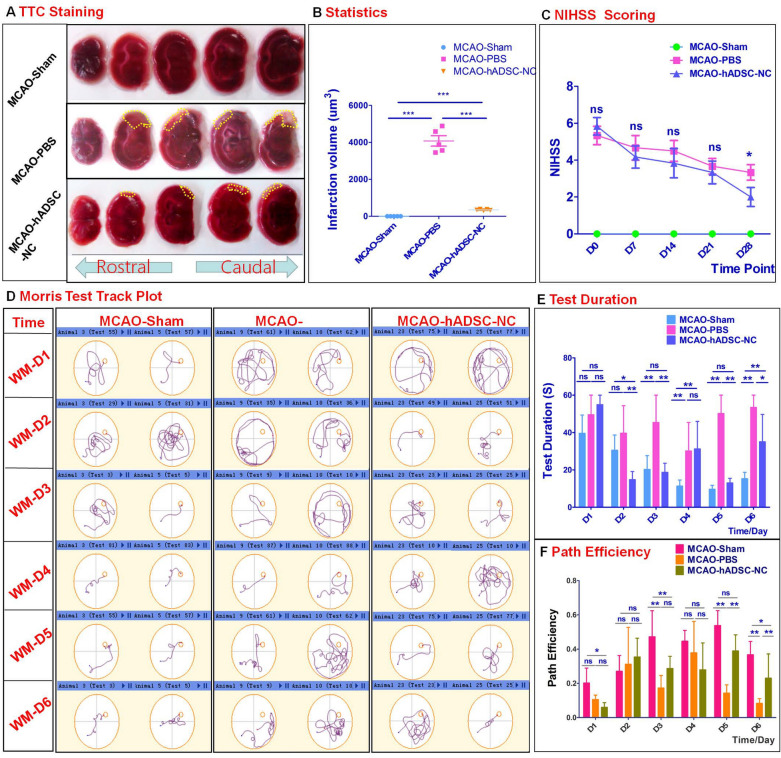
hADSC-NCs can decrease the MCAO mouse infarction volume and improve neurological scores, spatial learning and memory. **A** Shows TTC data to demonstrate the infarction volume variance among the groups. **B** shows the statistical analysis of the infarction volume. **C** Shows NIHSS data indicating the improvement in neurological behavior between the MCAO-PBS group and the MCAO-hADSC-NC group. **D** Compares the track plots from different groups at different time points. E shows the statistics for the test duration. F shows the path efficiency variance. n = 7. * indicates P < 0.05, ** indicates P < 0.001. *** indicates P < 0.0005

The MCAO reperfusion ischemic stroke mouse model was generated according to a previously described method [22]. Seven days after surgery, EGFP-labeled hADSC-NCs were stereotactically injected into the infarcted brain region of the MCAO mice, which were subjected to observation and evaluation for 4 weeks. In the PBS-treated control group, TTC staining revealed significant ischemic injury of the brain (grey areas in Fig. 2A), which was consistent with the abnormal behavior performance, as shown in Fig. 2C. Statistical analysis of the infarction volume is shown in Fig. 2B. The movement behaviors were observed in a double-blinded manner, and the NIHSS neurological score results are summarized in Fig. 2C. hADSC-NC transplantation led to a significant decrease in the infarction areas (Fig. 2A and B, P < 0.01) as well as the NIHSS scores (Fig. 2C) at 28 days after treatment. To evaluate the learning and memory ability of the MCAO mice, morris maze test was applied, and the movement tracks of the mice were monitored for six consecutive days (one program consisted of five training days plus one testing day). Representative tracks are shown for further analysis (Fig. 2D). hADSC-NC treatment significantly decreased the test duration (MCAO-hADSC-NCs: 35.03 ± 25.41 versus MCAO-PBS: 53.43 ± 11.37) and increased the path efficiency (MCAO-hADSC-NCs: 0.23 ± 0.24 versus MCAO-PBS: 0.08 ± 0.05) at day 6 compared with the PBS control mice (Fig. 2E and F, P < 0.05), although some differences still existed between the hADSC-NC-treated and sham groups. Our results indicate that hADSC-NC transplantation can partially facilitate the functional rescue of learning and memory in MCAO mice.

### Transplanted hADSC-NCs can migrate into various brain sites and survive for a long time after injection into the infarcted region in MCAO mice

**Fig. 3:**
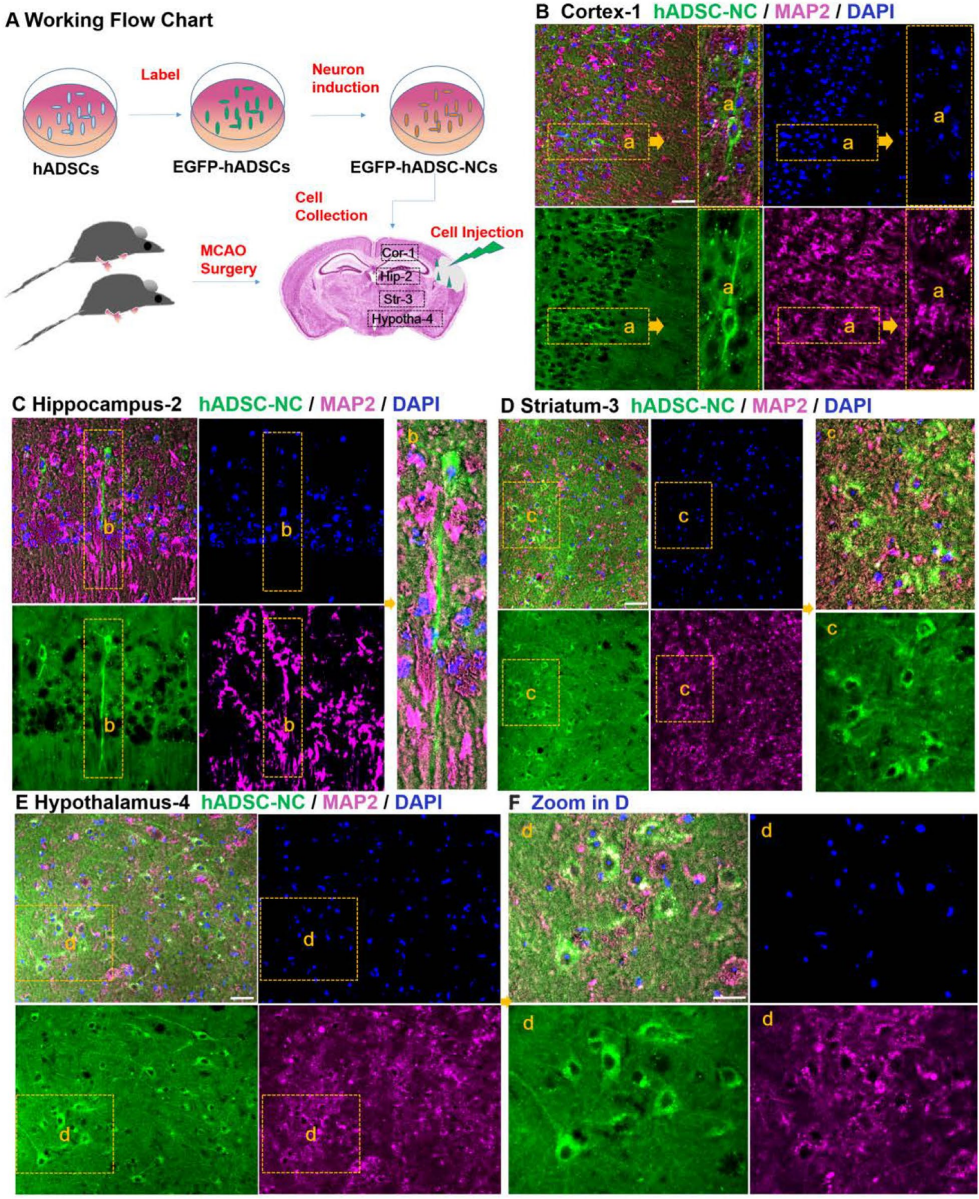
Tracking and identification of hADSC-NCs in MCAO mouse brains 4 weeks after transplantation. **A** Shows the working flowchart. Immunohistochemical staining data show that GFP and MAP2 double-positive hADSC-NC cells can be found in different areas of the mouse brain, including the cortex (**B**), hippocampus (**C**), striatum (**D**) and hypothalamus (**E**). Scale bar = 25 μm

The behavioral improvement resulting from hADSC-NC transplantation in the MCAO reperfusion mouse model leads us to postulate that these NCs might be able to escape immune rejection and survive injury in vivo. To track their committed fates of these transplanted cells, we labeled the hADSC-NCs with a lenti-EGFP-expressing virus in advance in vitro and immunostained the neuronal-specific marker MAP2 in different brain regions (Fig. 3A). To our surprise, the EGFP-labeled hADSC-NCs could be detected not only in the main infarction areas, such as the cortex and hippocampus, but also widely in other unaffected areas, including the striatum and hypothalamus (Fig. 3B–F). Most of the EGFP-labeled cells showed typical neuron morphology and MAP2 positive, implying their neuronal fate. Interestingly, even after 2 months, the transplanted human cells not only survived but also demonstrated more complex structures with extended dendrites and an increased number of branches (Fig. 3B and C, inserted panels), which suggested that given more time, the local microenvironment might have supported further transdifferentiation and maturation of the transplanted cells.

### hADSC-NCs directly contributed to spatial learning and memory recovery by improving LTP

**Fig. 4:**
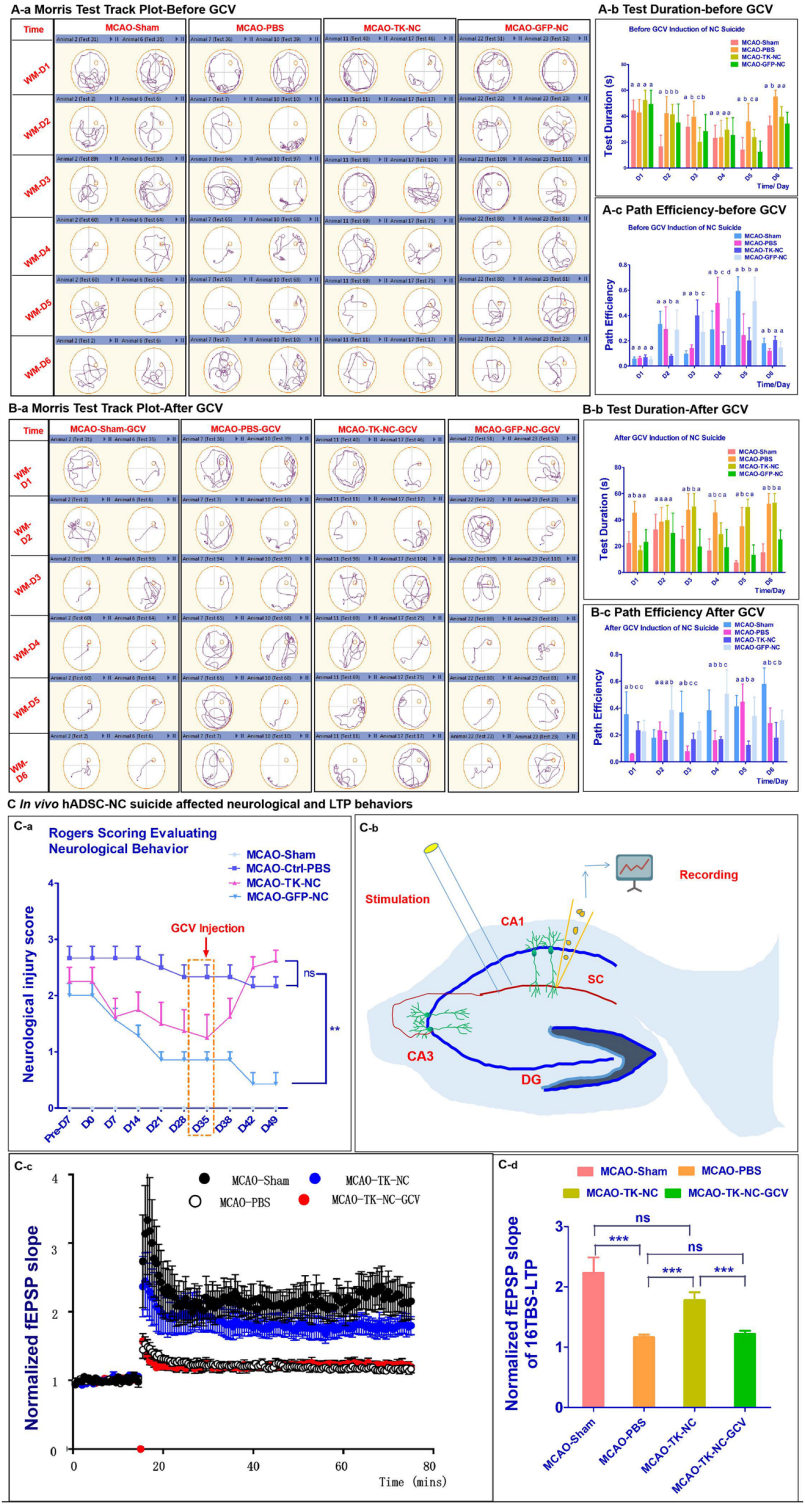
hADSC-NC suicide leads to the relapse of spatial learning and memory. **A** Show that hADSC-NCs rescued the brain function of MCAO mice according to the Morris test, **B** shows hADSC-NC suicide can lead to spatial learning and memory relapse after i.p. injection of ganciclovir (GCV), **a** Shows the track plots of representative mice from each group, **b** shows the test duration in each group, **c** shows the path efficiency of each group. **C** shows that hADSC-NC suicide increases the Rogers scores and decreases long-term potentiation (LTP). **C**-**a** shows the neurological evaluation in each group, **C**-**b** shows the schematic of LTP recording, **C**–**c** shows the TBS-induced CA3-CA1 LTP in each group, **C**-**d** shows a summary of the data on the magnitude of LTP observed in **C**-**c**. n = 7. * indicates P < 0.05, ** indicates P < 0.001, *** indicates P < 0.0005

Whether the transplanted hADSC-NCs could directly contribute to improved brain function has always been debated in the field of stem cell therapies, and the hypothesis of cell replacement or paracrine mechanisms may be favored. To clarify this issue, an in vivo cell suicide system was applied by infecting the transplanted hADSC-NCs with a thymidine kinase (TK) overexpression system and administering GCV intraperitoneally afterward to induce cell suicide in vivo [21]*.* Consistently, as shown in Fig. 4, the groups of mice transplanted with TK-NCs and GFP-NCs demonstrated significant improvements in spatial learning and memory compared with the PBS control group 4 weeks after establishing the MCAO models. In addition, the MCAO-TK-NC and MCAO-GFP-NC groups of mice showed similar spatial learning and memory abilities compared with the MCAO-Sham group of mice. Their representative swimming tracks are shown in Fig. 4A-a, and the test duration and path efficiency results for the Morris test are shown in Fig. 4A-b and Fig. 4A-c. After the first round of the Morris maze test, i.p. GCV administration was performed every day on every mouse in every group for later 1 week. At the end of GCV treatment, all groups of mice were subjected to the second round of the Morris maze test, and the data is shown in Fig. 4B. The swimming tracks are shown in Fig. 4B-a, and the test duration and path efficiency are shown in Fig. 4B-b and Fig. 4B-c, respectively. The track combined with test duration data showed that after 5-day training program, on the final test day 6, both hADSC-NC treated groups of MCAO-TK-NC and MCAO-GFP-NC achieved significant shorter (P < 0.05) tracks and higher (P < 0.05) path efficiency compared with MCAO-PBS group and comparable with MCAO-Sham group (Fig. 4A). The MCAO-TK-NC group of mice, in which the transplanted hADSC-NCs were induced to commit suicide by GCV treatment, showed significantly (P < 0.05) increased track length, test duration and decreased path efficiency, which indicated relapse in spatial learning and memory (Fig. 4B). The MCAO-GFP-NC group, as a parallel control with transplanted hADSC-NCs that survived without GCV treatment, showed similar spatial learning and memory capabilities with the MCAO-Sham mice, which greatly decreased the test duration and increased the path efficiency compared with the results of the first Morris maze test (Figure A&B). The Morris maze test results were consistent with the Rogers scoring data, as shown in Fig. 4C-a. GCV treatment on day 35, also leaded to the gradually relapsed movement functioning in the MCAO-TK-NC group. To further elucidate the electrophysiological basis for the improvement of spatial memory, hippocampal synaptic transmission was examined in the CA1 region of acute hippocampal slices from the MCAO-Sham, MCAO-PBS, MCAO-TK-NC and MCAO-TK-NC-GCV groups of mice. Field excitatory postsynaptic potentials (fEPSPs) in the CA1 region of the hippocampal slices were recorded in response to stimulation of the Schaffer collateral (SC) pathway. Long-term potentiation (LTP) of synaptic transmission, which is the major cellular mechanism that underlies learning and memory, was induced by tetanic stimulation. A schematic illustration of the experiment is shown in Fig. 4C-b. Hippocampal LTP was impaired in the MCAO-PBS group but rescued by transplantation of TK-NCs (MCAO-Sham: 223% ± 25% of the baseline; MCAO-PBS: 116 ± 4% of the baseline; MCAO-TK-NC: 178 ± 13% of the baseline; P < 0.001 for MCAO-Sham versus MCAO-PBS, P > 0.05 for MCAO-Sham versus MCAO-TK-NC, Fig. 4C-c and -d). However, the restoration of LTP in the MCAO-TK-NC group was abolished by i.p. GCV administration (MCAO-TK-NC-GCV: 122 ± 6% of the baseline, P < 0.001 for MCAO-TK-NC-GCV versus MCAO-TK-NC, Fig. 4C-c and -d), which implied that the TK-NCs directly participated in the reconstitution of neural circuits in the hippocampus.

### Transplanted hADSC-NCs grew into electrophysiologically active and healthy neuron cells in vivo

**Fig. 5:**
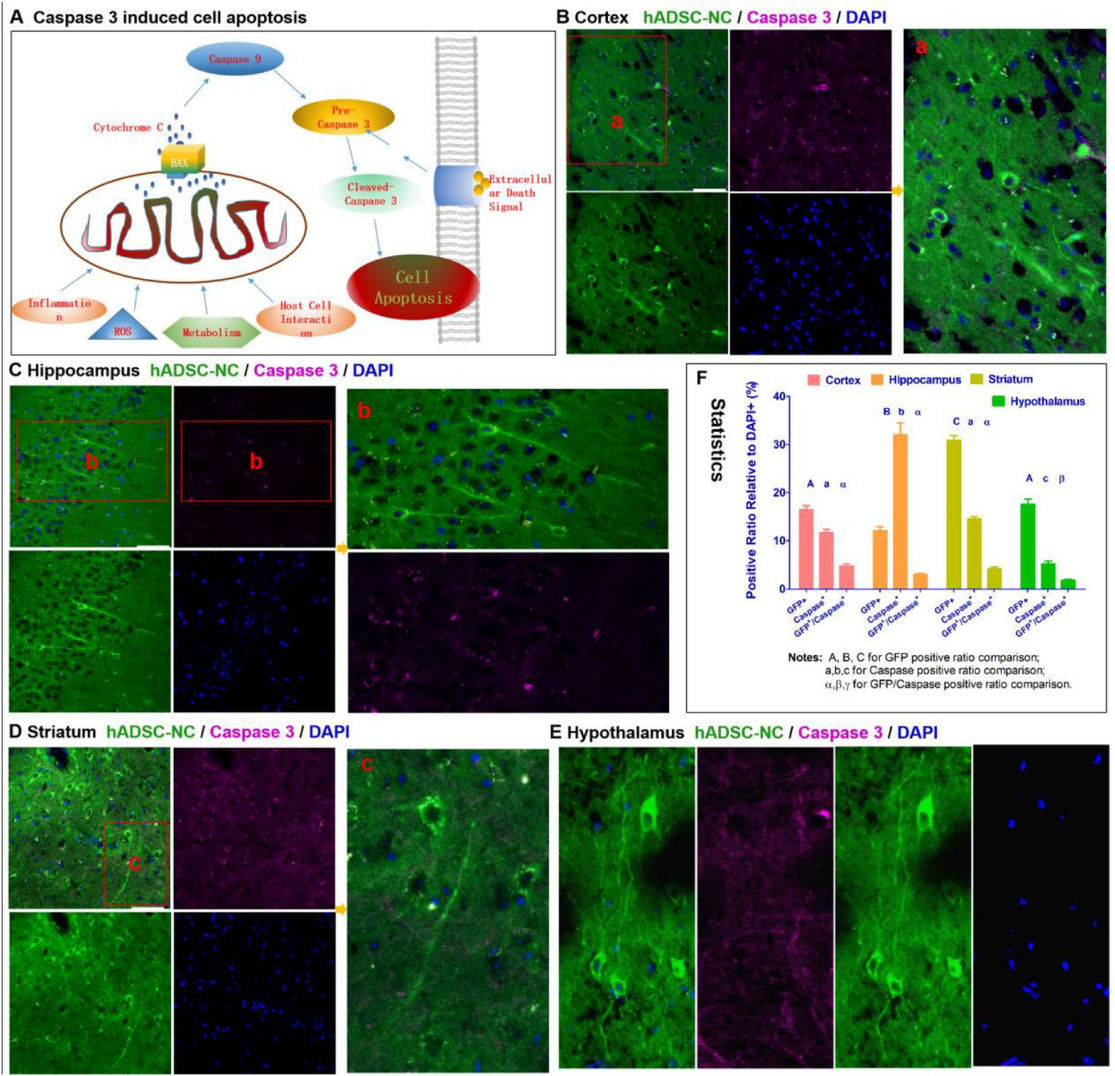
Characterization of hADSC-NCs in various MCAO mouse brain regions. **A** Shows the cell apoptosis marker cleaved Caspase 3. **B** Shows the negative expression of cleaved Caspase 3 in GFP-positive hADSC-NCs in the cortex. **C** Shows that the transplanted hADSC-NCs express cleaved Caspase 3, integrate well into the hippocampal tissue, and exhibit a healthy and complex neuron morphology. **D**, **E** Show similar cell survival, integration and neuron differentiation in the mouse striatum and hypothalamus. **F** Shows the statistical analysis of the hADSC-NC distribution, Caspase 3 positive percentage and GFP/Caspase 3 double-positive percentage. n = 5, Scale bar = 50 μm in **B**–**D**, scale bar = 25 μm in E

**Fig. 6:**
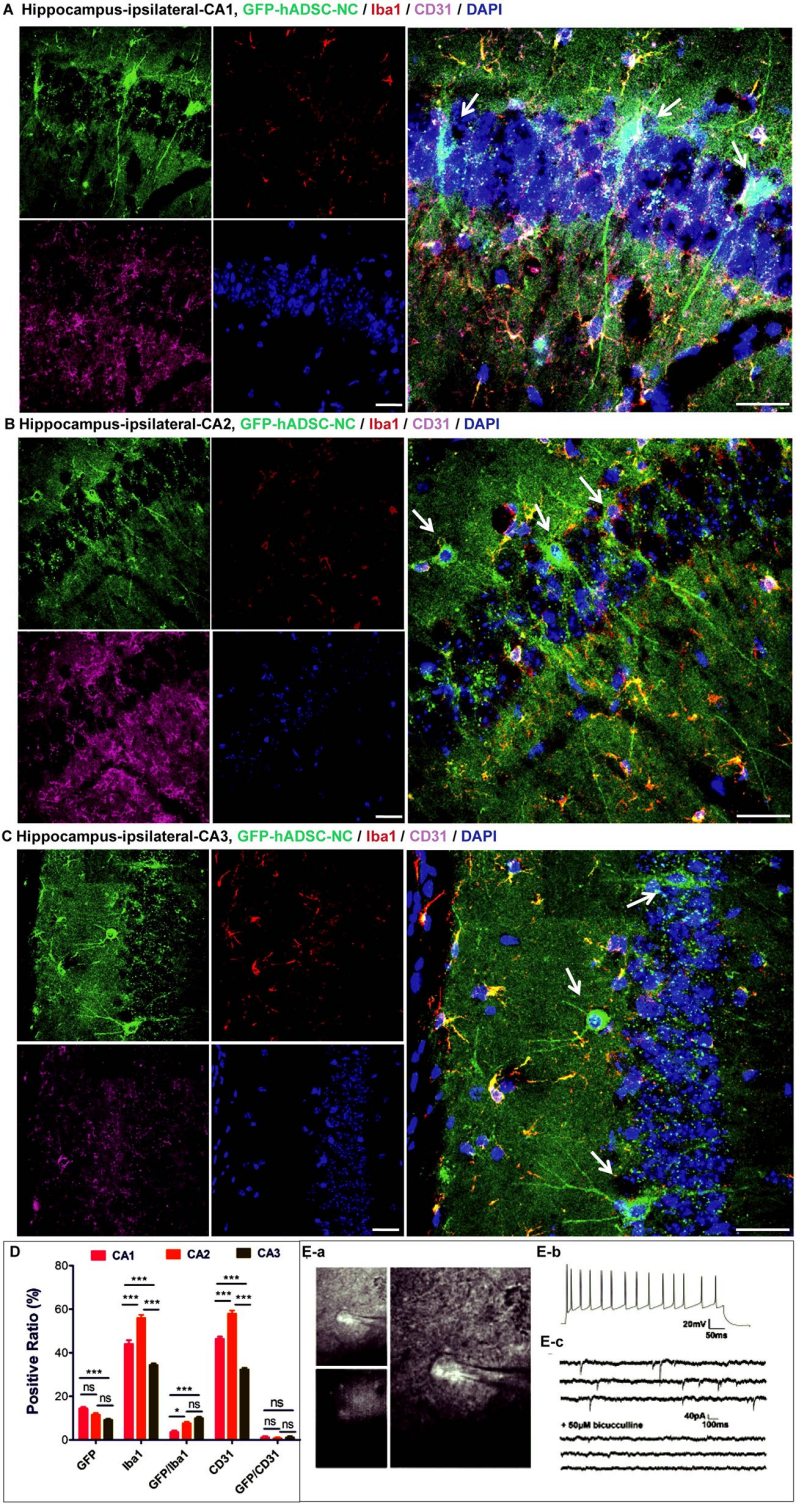
hADSC-NC terminal differentiation and brain vasculature rebuilding in the MCAO mouse hippocampus. The minus hADSC-NCs express the microglial cell marker Iba1 and the blood vessel marker CD31 in 3 areas of the hippocampus: **A** for CA1, **B** for CA2 and **C** for CA3. **D** shows the statistical analysis of the hADSC-NC distribution, the Iba1-positive percentage and the CD31-positive percentage in CA1, CA2 and CA3. E shows the electrophysiological properties of GFP-positive hADSC-NCs in live hippocampal slices. **a** Representative image of GFP-positive hADSC-NC-derived neurons during patch-clamp recording. **b** Action potentials could be elicited by a current injection. **c** Spontaneous IPSCs were recorded in patched neurons and blocked by 50 μM bicuculine. n = 7/8, Scale bar = 25 μm

To determine the fate of the transplanted cells, immunohistochemical staining was used to trace the migration, transdifferentiation and integration of the introduced hADSC-NCs in brain tissue. The staining results showed that most of the hADSC-NCs expressed the neuronal marker MAP2(Fig. 3), which is consistent with the in vitro cell staining (Fig. 1). GFP-positive neuron-like cells derived from hADSCs could always be observed in the cortex, hippocampus, striatum, hypothalamus, and other areas with different cell morphologies (Figs. 3 and 5). The GFP-positive hADSC-NCs survived well and expressed minor amounts of Caspase 3 (Fig. 5 and Additional file 1: Fig. S1), which indicated their health and excellent supportive functioning for endogenous neurons with high biological compatibility. Colocalization of human nuclear antigen (HuNA) and GFP could be observed, indicating that the GFP-positive cells were indeed the introduced human ADSC-NCs (Additional file 1: Fig. S1). HuNA & GFP double-positive, Caspase 3-negative hADSC-NCs were widely distributed within the CA1, CA2 and CA3 areas of the hippocampus, which predicted good spatial learning and memory recovery, as shown in Additional file 1: Fig. S1. Few GFP-positive hADSC-NCs expressed the microglial marker Iba1 and the blood vessel endothelial cell marker CD31, indicating that they did not transform into microglia or endothelial cells, as shown in Fig. 6A–D. The injected hADSC-NCs could transform into fully mature neuron cells with action potentials similar to those of endogenous neurons (Fig. 6E). The representative GFP-positive cell image obtained by whole-cell recording (Fig. 6E-a), with action potentials that were elicited by injection of current (Fig. 6E-b) and spontaneous postsynaptic currents that were blocked by bicuculline (50 μM) (Fig. 6E-c), reflecting the inhibitory (GABA) neurotransmission inputs in these neurons. These results also verified the hypothesis that the in vivo microenvironment may promote the differentiation of preconditioned immature hADSC-NCs (Fig. 1D-c) into fully mature neurons. These studies further proved that priming hADSCs to differentiate into neuron-like cells (hADSC-NCs) before transplantation is indeed an effective strategy because a high proportion of the hADSC-NCs survived and transdifferentiated into functional mature neurons rather than astrocytes, microglia or endothelial cells. The colocalization of Ki67 and GFP was also randomly observed, indicating that some of the transplanted hADSC-NCs still maintained proliferative ability in vivo* (*Additional file 1: Fig. S2).

### hADSC-NCs exerted bidirectional inflammatory effects by suppressing glial cell overreaction and augmenting the expression of pro- or anti-inflammatory factors

**Fig. 7:**
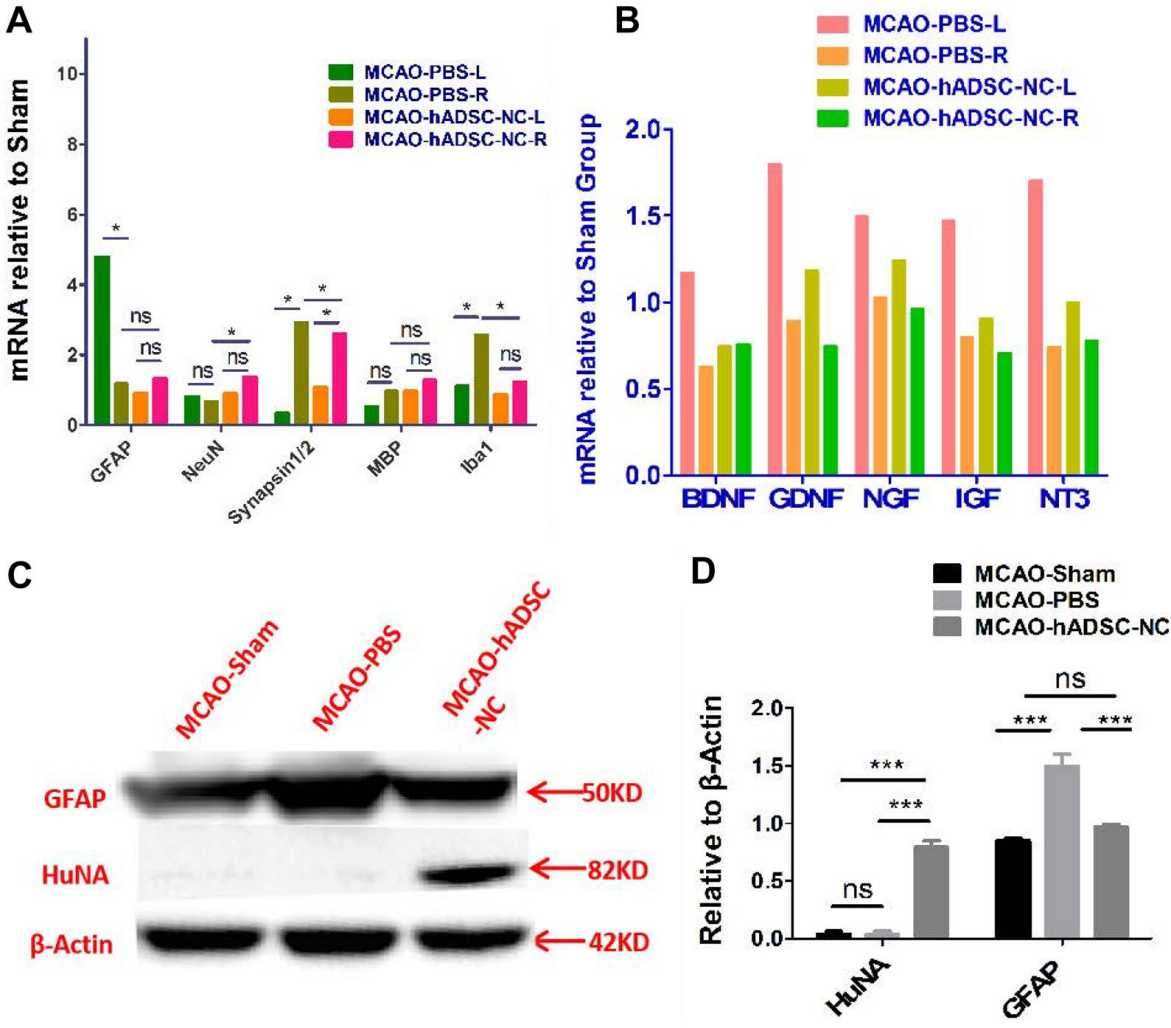
Molecular analysis to demonstrate the interaction between hADSC-NCs and brain host cells as well as the effects on neurotrophic factor secretion. **A** Show that hADSC-NCs affected the mRNA expression of the neuronal markers NeuN, synapsin 1/2, microglia marker Iba1 and astrocyte or neuronal stem cell marker GFAP. **B** Shows that hADSC-NCs scarcely changed the expression of neurotrophic factors at the mRNA level. **C** Shows that GFAP expression was inhibited by hADSC-NCs; HuNA could be detected in the mouse brain in the MCAO-hADSC-NC group, but HuNA could not be detected in the MCAO-Sham or the MCAO-PBS group by western blotting

Since natural ADSCs can exert positive immune modulation, it is of great interest to test whether the transplanted hADSC-NCs still maintain this capacity. qRT-PCR and western blotting data indicated that hADSC-NC administration significantly suppressed the expression of the microglial marker Iba1 (P < 0.01) of the ipsilateral (Right) brain (Fig. 7A) and astrocyte marker GFAP (P < 0.05) of total brain (Fig. 7C and D) compared with that in the PBS control. This indicated that hADSC-NCs could decrease brain inflammation by suppressing the activation of microglia and astrocytes induced by brain ischemic reperfusion injury. On the other hand, hADSC-NCs significantly (P < 0.05) enhanced the expression of neuronal genes, such as NeuN and Synapsin 1/2, in the host brain (Fig. 7A) compared with that in the PBS-injected control group mice. This implied that the introduced hADSC-NCs may protect endogenous neurons from death. However, the expression of the oligodendrocyte marker MBP was not significantly (P > 0.05) increased by hADSC-NCs compared with its expression in the PBS control group. In addition, no apparent differences in the expression of neurotrophic factors, including BDNF, GDNF, NGF, IGF and NT3, were observed between the ipsilateral brain spheres of the PBS and hADSC-NC groups, which demonstrated that the paracrine function of ADSC-NCs in boosting neurotrophic factors might not play a vital role in stroke rescue (Fig. 7B). Western blotting experiments further confirmed that GFAP expression could be suppressed after hADSC-NC transplantation, and the human-specific marker HuNA could be detected, as shown in Fig. 7C.

How the transplanted hADSC-NCs affected the profiles of local and systemic chemokines and cytokines was further explored by protein microarray panel. The data demonstrated that hADSC-NCs exhibited bidirectional local immune regulation ability by suppressing the pro-inflammatory factors IL-1α, IL-1β, IL-2 (P < 0.05), and MIP-1β (P < 0.05) meanwhile promote pro-inflammatory factors of IP-10 (P < 0.05) MCP-1 (P < 0.05) and enhancing the activity of the anti-inflammatory factors IL-15 in the collected brain tissue, as shown in Additional file 1: Fig. S3A&B. Meanwhile, only two proinflammatory factors, IL-1α and KC, were downregulated in the blood serum samples, as shown in Additional file 1: Fig. S3C&D. These results indicated that hADSC-NCs could still exert inflammatory modulation in the local injured sites of the brain while inducing only a minor systemic immune response.

## Discussion

Mesenchymal stem cell (MSC) therapy is becoming a promising new therapeutic option for stroke. Of all types of MSCs, ADSCs are of special interest based on recent data from both animal and clinical studies [4, 13, 22, 51]. Previous reports demonstrated the feasibility of the transdifferentiation of hADSCs into neuron-like cells [20, 21, 49, 67]. Our data indicated that hADSCs could be efficiently transdifferentiated into immature neuron-like cells with synaptic activities, which is consistent with the results of the most recent reports from other research groups [3, 25, 49]. hADSCs could be gradually induced to differentiate into GFAP-negative and MAP2-, SYNAPSIN 1/2-, NeuN- and vGLUT-positive neuron-like cells (hADSC-NCs), which can be stained at different time points. To partially maintain the proliferative ability of hADSC-NCs and improve their survival after transplantation into the brain, we pre-treated hADSCs according to our neuronal induction protocol for 24 h before transplantation. Transplantation of these pre-conditioned cell populations could significantly enhance the spatial learning and memory of MCAO reperfusion mice in the sub-acute phase, which may achieve better therapeutic effects compared with intravenous transplantation [7, 22]. As expected, a decrease in the test duration and an increase in path efficiency were found in the hADSC-NC group compared with the PBS group of MCAO mice, indicating neurological improvement due to hADSC-NC transplantation. The infarction volume was also decreased in the hADSC-NC group compared with the PBS group. These data demonstrated that intracerebral transplantation of hADSC-NCs could dramatically rescue the neurological function of MCAO mice.

Although current data on the efficacy of stroke treatment with hADSCs show obvious discrepancies due to the use of different animal models, various cell delivery routines and time windows of cell administration, therapeutic effects could be consistently observed in most animal experiments when using hADSCs [22, 23, 42, 72]. However, how transplanted hADSCs achieve the expected long-term therapeutic effects has rarely been explored in depth until now. Whether the transplanted cells could exert treating effects through cell replacement therapies kept elusive yet. Therefore, verifying the survival, differentiation, and integration of hADSCs in vivo as well as their interaction with host resident cells has become necessary and urgent. This study demonstrated that the introduced hADSC-NCs could survive well for up to 6 months and fully integrate into endogenous tissue, which led to the repair of infarcted brain regions. The colocalization of EGFP and MAP2 was found at various brain sites, including the cortex, hippocampus, striatum and hypothalamus. Minor percentages (< 10.0%) of cells with EGFP and Iba1 colocalization were found. The EGFP-labeled hADSC-NCs migrated to almost all areas of the brain with typical neuronal morphology. Action potentials and synaptic activities were recorded by whole-cell patch-clamp experiments in acute brain slices with EGFP-labeled hADSC-NCs, and these could be blocked by bicuculline, reflecting the presence of inhibitory (GABA) neurotransmission inputs in these neurons. This may be the prerequisite for their functional involvement in the endogenous neural circuit. These data indicated that in vitro primed hADSC-NCs could be further transdifferentiated into fully mature neuron cells with electrophysiological activities within the brain microenvironment, which strongly proved the feasibility of hADSC transdifferentiation into mature neuron cells and the possibility of their use for cell replacement in vivo for the treatment of traumatic or degenerative neural diseases. Compared with the PBS group, the hADSC-NC group showed significant improvement of LTP to a similar level as that in the Sham group. After hADSC-NCs committed suicide, the ability to induce LTP was largely abolished, which was consistent with the results of the Morris maze test and Rogers scoring and the NIHSS data. In combination, these data strongly suggest that hADSC-NCs may directly participate in neural circuit rebuilding. To the best of our knowledge, this is the first report showing that hADSCs can differentiate into electrophysiologically active neurons in vivo after transplantation and participate in the reconstitution of neural circuits for stroke treatment.

The immune modulatory capacity of hADSCs has been broadly reported and has a profound impact on their therapeutic effect [11, 41, 71]. Whether the primed intermediate hADSC-NCs still show these characteristics has not been thoroughly explored, though it was implied by previously published data [14]. This study demonstrated that hADSC-NCs exerted significant bidirectional local immune modulatory effects by suppressing pro-inflammatory factors IL-1α, IL-1β, IL-2 and MIP-1β and promoting proinflammatory factors IP-10, MCP-1, meanwhile promote anti-inflammatory factor of IL-15. At the same time, no significant differences in the systemic serum levels were detected, except for the minor downregulation of the pro-inflammatory factors of IL-1α and KC in serum, which indicates that in situ transplantation of hADSC-NCs scarcely induced systematic immune responses. Our data suggest that hADSC-NCs may maintain their positive immune modulation effects locally while showing low systemic immunogenicity. It has been widely accepted that hADSCs can promote host cell survival and inhibit neuronal apoptosis [35, 44, 50]. It is of interest to know whether hADSC-NCs still possess these capabilities when used to treat traumatic neural diseases. Our data indicated that hADSC-NCs could protect the survival of endogenous neurons from hypoperfusion damage presumably by suppressing the activation of GFAP- and Iba1-positive glia cells other than increasing neurotrophic factor expression.

In summary, this study demonstrates that the brief priming of hADSCs under neuronal differentiation conditions in vitro will significantly enhance their neuronal fate in vivo in an MCAO reperfusion mouse model, augment their viability and facilitate their migration, tissue integration and functional maturation. Given time, these transplanted hADSC-NCs will become more mature in terms of their electrophysiological activity and become capable of firing action potentials and exhibiting synaptic activities. More importantly, they will directly participate in rebuilding neural circuits and play a pivotal role in long-term synaptic plasticity, which will eventually lead to the improvement of spatial learning and memory in MCAO reperfusion mice. Although the detailed mechanism of how the endogenous environment affects the fate of these preconditioned hADSCs is yet to be revealed, our study provides a new perspective for the development of a novel therapeutic strategy to combat stroke, a devastating disease that affects millions of people worldwide. Though with some limitations including being scarce of experiments in non-human primates and deeper exploration of hADSC-NCs on immue modulation, this study throws some lights on further studies on cell replacement therapies with hADSCs in treating neuron degenerative diseases.

## Experimental procedures

All animal experiments were performed according to protocols approved by the Ethical Committee of the Experimental Animal Center affiliated with the School of Medicine of Tongji University. The approval no. is TJLAC-014-012. hADSCs were isolated from human adipose liposuction liquid, and the processing and handling protocols were approved by the Ethical Committee of East Hospital Affiliated with Tongji University. The committee director is Fu, Meng. The approval no. is 2015-045. The human adipose liposuction liquid was obtained from the cosmetic plastic surgery hospital with the consent of patients.

### Obtaining electrophysiologically active hADSC-NCs and characterization

hADSCs cultured on Matrigel-coated coverslips in 24-well plates were induced to differentiate into neural-like cells with neural induction cocktail medium according to our previous publication[20] with minor modifications. The stepwise induction protocol is described in Fig. 1D-a. Cytoimmunostaining was performed to characterize the hADSC-NCs [20]. Whole-cell patch clamping was used to determine the electrophysiological properties.

### Middle cerebral artery occlusion (MCAO) mouse model

The MCAO mouse was generated through surgery on C57/BL6 mice according to the procedure described by Zhou et al. [72] Briefly, after inducing anesthesia, a small incision was made above the rhinal fissure to expose and isolate the right common cerebral artery (CCA) and the branch of the cerebral artery. The CCA branch was permanently ligated just before its bifurcation into the frontal and parietal branches with a 9–0 suture. The external common carotid artery was then permanently ligated at two sites and cut in the middle of the two sites. The internal common carotid artery was temporarily ligated. A breach was made between the bifurcation and the ligation site in the middle cerebral artery to allow the embolus (Cat#2634-A4, Beijing Sunbio Biotech Co., Ltd) to be infixed into the internal common carotid artery, reaching a depth of approximately 18 mm. One hour of occlusion followed by reperfusion was achieved by carefully removing the embolus to avoid bleeding. Then, stitching and necessary sterilization were performed. Mice that received surgery were evaluated for their neurological function according to the Rogers scale score system or the NIHSS [13, 43, 57]. Successful MCAO mice with scores between 2 and 3 were randomly assigned to the PBS control group (MCAO-Ctrl-PBS) or the hADSC-NC injection group (MCAO-hADSC-NC). To confirm the safety of the surgery, a sham group (MCAO-Sham), which was subjected to blood vessel exposure without occlusion, was also included as the normal control.

### hADSC-NC labeling, preparation and stereotactic injection into the MCAO brain injury area

To obtain the transplanted hADSC-NCs, hADSCs were first labeled with an EGFP-expressing lentivirus or mCherry-TK-overexpressing lentivirus. Forty-eight hours after infection, hADSCs were observed under a fluorescence microscope at wavelengths of 488 nm and 568 nm. A percentage of hADSCs labeled with green fluorescence of 80–90% indicated successful infection. Then, the labeled hADSCs were incubated with the aforementioned modified cocktail [20, 61], and induced to differentiate into neuron-like cells for 24 h. hADSC-NCs were collected and resuspended at a final concentration of 1 × 109 cells/ml in PBS. Five microliters of this cell suspension were injected into the MCAO mouse brain injury lesion through a glass micropipette and stereotaxic injector (KDS310, Muromachi-Kikai). The detailed procedure was similar to our previously published procedure [72].

### Neurological function evaluation according to the Rogers scaling system and the NIHSS

Each mouse was neurologically evaluated according to the Rogers scaling system by two researchers who were blinded to the experimental group assignments. The scale categories used were previously reported [72]. The NIHSS system was used as described in the reference [13, 61]. Scoring was performed every week, and the statistical analysis was carried out with GraphPad Prism 5. Each group had at least 7 mice.

### Mouse brain infarction volume determined by TTC staining

TTC staining was performed as previously described [72]. After the mice were sacrificed, the cerebrum was immediately removed and placed in a − 4 °C refrigerator for 20 min. Then, it was sliced into six uniform coronal sections after the olfactory bulb was removed. The sections were placed in 2% (W/V in PBS) 2,3,5-triphenyltetrazolium chloride (TTC, Cat#T8877, Sigma) at 37 °C in a water bath and then fixed with 4% paraformaldehyde. The normal brain tissue was dyed pink, while the infarction area was gray. The data processing was performed according to previously published methods [72].

### Mouse spatial learning and memory evaluation with the Morris test

To evaluate the effect of hADSC-NCs on mouse spatial learning and memory, a Morris water maze equipped with a digital camera was used to determine the physiological indexes for the poststroke mice. The general protocols were similar to those previously published with minor modifications [12, 53]. The Morris test program consisted of 5 days of training plus 1 final test day. The data were recorded and processed with Any-maze software (EthoVision XT7.0, Noldus Information Technology b.v., Netherlands).

### Immunohistochemical staining to trace hADSC-NC fate

The EGFP-hADSC-NCs were tracked and identified by immunohistochemical staining with the neuron markers MAP2 (Synaptic Systems, Cat#188011 and Cat#188003), NF-200 (Cell Signaling, Cat# 2836), NeuN (Sigma, Cat# SAB4300883), Synapsin1/2 (Cell Signaling, Cat#5297) Human Nuclear Antigen (HuNA, Merck Millipore, Cat# MAB1281), the proliferative marker Ki67 (R&D Systems, Cat#AF7649), astrocyte marker of GFAP (Synaptic Systems, Cat#173011), microglia marker of IbaI (FUJI FILM, Cat# 019-19741), the cell apoptosis marker Caspase 3 (Cell Signaling, Cat# 9579S) and blood vessel endothelial marker of CD31(R&D Systems, Cat#AF3628) by following previously published methods [72]. Briefly, after the Morris water maze test, mice from each group were sacrificed, and intracardial perfusion was performed. Then, the brains were removed, fixed with 4% paraformaldehyde and dehydrated with a sucrose gradient. Brain sections 20 μm in thickness were obtained with a cryostat (Leica, CM1850), followed by immunohistochemical staining. The distribution of the EGFP-labeled hADSC-NCs in specific brain sites was observed through confocal microscopy (Leica, SP8). Additionally, colocalization was carefully observed to determine whether any transdifferentiation of hADSC-NCs into neuronal cells had occurred.

### HSV-TK-mCherry-GCV cell suicide system construction and application

The HSV-TK-mCherry packaging system was provided by our colleague Prof. Zhang Hongsheng. The in vitro infection of hADSCs was first performed to determine the appropriate GCV amount for in vivo i.p injection. Five weeks after HSV-TK-mCherry-hADSC-NC transplantation, all groups received a 7-day i.p. injection of ganciclovir (GCV, Sigma-Aldrich, Cat# G2536) at 100 ng/kg body weight per day to induce the introduced cells to commit suicide by following previously published method [21, 60]. The Rogers scores, Morris test results and field potentials of the acute brain slices were all determined before and after GCV injection in each group of mice according to the method in a previous publication [18].

### Determination of brain neuronal markers and neurotrophic factor variation by qRT-PCR and western blotting

Total mRNA was extracted and purified by using a TRIzol Reagent Kit (Invitrogen, Cat#15596-018) and then subjected to reverse transcription into cDNA as a template for qRT-PCR (Invitrogen, Cat#11731-015). To further explore whether the introduced hADSC-NCs efficiently inhibited astrocyte reactions, western blotting was used to detect the astrocyte marker GFAP in different groups by following standard protocols with the house keeping antibody of β-Actin( Cell Signaling, Cat#8457) [21]. Furthermore, the human nuclear-specific marker HuNA was also detected to confirm whether hADSC-NCs specifically migrated to the brain. The protocols were previously published [21].

### Electrophysiological activity determination for cultured hADSC-NCs and live brain slices

Coronal hippocampal slices (400 μm) were prepared and maintained in Artifical Cerebrospinal Fluid(ACSF) containing (in mM) NaCl (119), NaHCO_3_ (26.2), NaH_2_PO_4_ (1), KCl (2.5), CaCl_2_ (2.5), MgSO_4_ (2.5) and D-glucose (11) at 25 °C for at least 1 h before use according to published protocols with modifications [26, 28, 29]. In brief, for the LTP experiments, the field Excitatory Postsynaptic Potential (fEPSPs) were recorded using 1.5–3.5 MΩ glass pipettes filled with ACSF that were placed in the stratum radiatum of the CA1 region. fEPSPs were evoked by stimulation of the Schaffer collateral pathway once every 30 s with a bipolar platinum electrode. LTP was induced by 16 bursts of stimulation (each burst consisting of 4 pulses at 100 Hz) delivered at 5 Hz (theta-burst stimulation, TBS). Baseline responses were recorded for at least 20 min. Responses were subsequently recorded for an additional 60 min after TBS. The magnitude of LTP was quantified as the normalized average slope of the fEPSP obtained from the last 15 min of recording.

For the whole-cell recordings in brain slices, the pipette resistance was 3–5 MΩ, and the internal solution contained (in mM) 135 KCl, 10 HEPES, 1 EGTA, 4 Mg-ATP, and 0.4 Na-GTP (pH adjusted to 7.4 with KOH). Action potentials were elicited by 500 ms of depolarizing current pulses ranging from 100 to 1000 pA in 100-pA increments. For the in vitro cultured hADSC-NCs, whole-cell recordings were obtained by following similar protocols [21, 63, 64].

### Immune factor profiling

To verify whether the introduced hADSC-NCs played any immune modulatory roles or had any kind of immune modulatory functions, 32 inflammation- or immune-related factors were placed in one 96-well plate to generate the mouse cytokine/chemokine magnetic bead panel. The protocols strictly followed the instructions obtained from the kit (MCYTOMAG-70K-PX32, Lot#2618731). The experiments were performed as a service provided by Merck Millipore.

### Statistical analyses

All data are expressed as the mean ± SD or SEM. The number of mice in each group was at least 7, and all the results were obtained from at least 5 replicates. The statistical significance of differences between groups was determined using one-way or two-way ANOVA with Bonferroni posttests with GraphPad Prism version 5.00 for Windows (GraphPad Software, San Diego California USA, www.graphpad.com). P < 0.05 was considered statistically significant.

## Supplementary Information

**Additional file 1.** Additional figures.

## Data Availability

The raw/processed data required to reproduce these findings cannot be shared at this time due to technical or time limitations. De-identified data are available from the corresponding author upon reasonable request after article published.

## Abbreviations

ACSF: Artifical Cerebrospinal Fluid
APC: Allophycocyanin
BDNF: Brain-derived neurotrophic factor
BMSCs: Bone Marrow Stem Cells
EGFP: Enhanced Green Fluorescence Protein
FACS: Fluorescent Activated Cell Sorting
fEPSPs: Excitatory Postsynaptic Potential
FITC: Fluorescein Isothiocyanate
GDNF: Glial cell line-derived neurotrophic factor
GFAP: Glial Fibrillary Acidic Protein
hADSCs: Human adipose-derived stem cells
hADSC-NCs: hADSC-derived neuron-like cells
IGF-1: Insulin-like Growth Factor-1
LTP: Long term potentiation
MAP2: Microtubule-Associated Protein 2
MCAO: Middle Cerebral Artery Occlusion
MSCs: Mesenchymal Stem Cells
NGF: Nerve growth factor
NT3: Neurotrophin 3
PE: Phycoerythrin
rtPA: Recombinant Tissue Plasminogen Activator
VEGF: Vascular Endothelial Growth Factor

## References

[1] Abdanipour A, Tiraihi T, Delshad A (2011). Trans-differentiation of the adipose tissue-derived stem cells into neuron-like cells expressing neurotrophins by selegiline. Iran Biomed J.

[2] Albers GW, Diener HC, Grind M, Halperin JL, Horrow J, Olsson SB, Petersen P, Vahanian A, Frison L, Nevinson M (2003). Stroke prevention with the oral direct thrombin inhibitor ximelagatran compared with warfarin in patients with non-valvular atrial fibrillation (SPORTIF III): randomised controlled trial. Lancet.

[3] Blecker D, Elashry MI, Heimann M, Wenisch S, Arnhold S (2017). New insights into the neural differentiation potential of canine adipose tissue-derived mesenchymal stem cells. Anat Histol Embryol.

[4] Burrow KL, Hoyland JA, Richardson SM (2017). Human adipose-derived stem cells exhibit enhanced proliferative capacity and retain multipotency longer than donor-matched bone marrow mesenchymal stem cells during expansion in vitro. Stem Cells Int.

[5] Chen AZ, Liu N, Huang H, Lin FF, Liu DS, Lin XH (2011). Outgrowth of neuronal axons on adipose-derived stem cell transplanting for treatment of cerebral infarction in rats. Chin J Cell Mol Imm.

[6] Chen JY, Gu ZJ, Wu MX, Yang Y, Zhang JH, Ou JS, Zuo ZY, Wang JF, Chen YX (2016). C-reactive protein can upregulate VEGF expression to promote ADSC-induced angiogenesis by activating HIF-1 alpha via CD64/PI3k/Akt and MAPK/ERK signaling pathways. Stem Cell Res Ther.

[7] Cheng F, Lu XC, Hao HY, Dai XL, Da Qian T, Huang BS, Tang LJ, Yu W, Li LX (2014). Neurogenin 2 converts mesenchymal stem cells into a neural precursor fate and improves functional recovery after experimental stroke. Cell Physiol Biochem.

[8] Chi K, Fu RH, Huang YC, Chen SY, Hsu CJ, Lin SZ, Tu CT, Chang LH, Wu PA, Liu SP (2018). Adipose-derived stem cells stimulated with *n*-butylidenephthalide exhibit therapeutic effects in a mouse model of parkinson's disease. Cell Transplant.

[9] Ciervo Y, Ning K, Jun X, Shaw PJ, Mead RJ (2017). Advances, challenges and future directions for stem cell therapy in amyotrophic lateral sclerosis. Mol Neurodegener.

[10] Danielyan L, Schafer R, von Ameln-Mayerhofer A, Bernhard F, Verleysdonk S, Buadze M, Lourhmati A, Klopfer T, Schaumann F, Schmid B (2011). Therapeutic efficacy of intranasally delivered mesenchymal stem cells in a rat model of Parkinson disease. Rejuv Res.

[11] De Miguel MP, Fuentes-Julian S, Blazquez-Martinez A, Pascual CY, Aller MA, Arias J, Arnalich-Montiel F (2012). Immunosuppressive properties of mesenchymal stem cells: advances and applications. Curr Mol Med.

[12] Diederich K, Schmidt A, Strecker JK, Schabitz WR, Schilling M, Minnerup J (2014). Cortical photothrombotic infarcts impair the recall of previously acquired memories but spare the formation of new ones. Stroke.

[13] Diez-Tejedor E, Gutierrez-Fernandez M, Martinez-Sanchez P, Rodriguez-Frutos B, Ruiz-Ares G, Lara ML, Gimeno BF (2014). Reparative therapy for acute ischemic stroke with allogeneic mesenchymal stem cells from adipose tissue: a safety assessment a phase II randomized, double-blind, placebo-controlled, single-center, pilot clinical trial. J Stroke Cerebrovasc.

[14] Du WJ, Reppel L, Leger L, Schenowitz C, Huselstein C, Bensoussan D, Carosella ED, Han ZC, Rouas-Freiss N (2016). Mesenchymal stem cells derived from human bone marrow and adipose tissue maintain their immunosuppressive properties after chondrogenic differentiation: role of HLA-G. Stem Cells Dev.

[15] Dulamea AO (2015). The potential use of mesenchymal stem cells in stroke therapy-from bench to bedside. J Neurol Sci.

[16] Duma C, Kopyov O, Kopyov A, Berman M, Lander E, Elam M, Arata M, Weiland D, Cannell R, Caraway C (2019). Human intracerebroventricular (ICV) injection of autologous, non-engineered, adipose-derived stromal vascular fraction (ADSVF) for neurodegenerative disorders: results of a 3-year phase 1 study of 113 injections in 31 patients. Mol Biol Rep.

[17] Ehrenreich H, Weissenborn K, Prange H, Schneider D, Weimar C, Wartenberg K, Schellinger PD, Bohn M, Becker H, Wegrzyn M (2009). Recombinant human erythropoietin in the treatment of acute ischemic stroke. Stroke.

[18] Falnikar A, Li K, Lepore AC (2015). Therapeutically targeting astrocytes with stem and progenitor cell transplantation following traumatic spinal cord injury. Brain Res.

[19] Feng NH, Jia YJ, Huang XX (2019). Exosomes from adipose-derived stem cells alleviate neural injury caused by microglia activation via suppressing NF-kB and MAPK pathway. J Neuroimmunol.

[20] Gao S, Zhao P, Lin C, Sun YX, Wang YL, Zhou ZC, Yang DJ, Wang XL, Xu HZ, Zhou F (2014). Differentiation of human adipose-derived stem cells into neuron-like cells which are compatible with photocurable three-dimensional scaffolds. Tissue Eng Pt A.

[21] Gao SN, Guo XX, Zhao SM, Jin YP, Zhou F, Yuan P, Cao LM, Wang J, Qiu Y, Sun CX (2019). Differentiation of human adipose-derived stem cells into neuron/motoneuron-like cells for cell replacement therapy of spinal cord injury. Cell Death Dis.

[22] Gomez-de Frutos MC, Laso-Garcia F, Diekhorst L, Otero-Ortega L, Fuentes B, Jolkkonen J, Detante O, Moisan A, Martinez-Arroyo A, Diez-Tejedor E (2019). Intravenous delivery of adipose tissue-derived mesenchymal stem cells improves brain repair in hyperglycemic stroke rats. Stem Cell Res Ther.

[23] Gon B, Dong YP, He C, Jiang WW, Shan Y, Zhou BY, Li WF (2019). Intravenous transplants of human adipose-derived stem cell protect the rat brain from ischemia-induced damage. J Stroke Cerebrovasc.

[24] Guo XL, Wang X, Li Y, Zhou B, Chen WD, Ren LH (2019). Olfactory ensheathing cell transplantation improving cerebral infarction sequela: a case report and literature review. J Neurorestoratology.

[25] Han C, Zhang L, Song L, Liu Y, Zou W, Piao H, Liu J (2014). Human adipose-derived mesenchymal stem cells: a better cell source for nervous system regeneration. Chin Med J-Peking.

[26] Hao R, Qi Y, Hou DN, Ji YY, Zheng CY, Li CY, Yung WH, Lu B, Huang Y (2017). BDNF val66met polymorphism impairs hippocampal long-term depression by down-regulation of 5-HT3 receptors. Front Cell Neurosci.

[27] Huang HY, Chen L, Mao GS, Sharma HS (2020). Clinical neurorestorative cell therapies: developmental process, current state and future prospective. J Neurorestoratology.

[28] Huang Y, Ko H, Cheung ZH, Yung KKL, Yao T, Wang JJ, Morozov A, Ke Y, Ip NY, Yung WH (2012). Dual actions of brain-derived neurotrophic factor on GABAergic transmission in cerebellar Purkinje neurons. Exp Neurol.

[29] Huang Y, Yoon K, Ko H, Jiao S, Ito W, Wu JY, Yung WH, Lu B, Morozov A (2016). 5-HT3a receptors modulate hippocampal gamma oscillations by regulating synchrony of parvalbumin-positive interneurons. Cereb Cortex.

[30] Imamura H, Adachi T, Kin T, Ono S, Sakai Y, Adachi T, Soyama A, Hidaka M, Takatsuki M, Shapiro AMJ (2018). An engineered cell sheet composed of human islets and human fibroblast, bone marrow-derived mesenchymal stem cells, or adipose-derived mesenchymal stem cells: an in vitro comparison study. Islets.

[31] Jahan R, Saver JL, Schwamm LH, Fonarow GC, Liang L, Matsouaka RA, Xian Y, Holmes DN, Peterson ED, Yavagal D (2019). Association between time to treatment with endovascular reperfusion therapy and outcomes in patients with acute ischemic stroke treated in clinical practice. Jama-J Am Med Assoc.

[32] Jeong CH, Kim SM, Lim JY, Ryu CH, Jun JA, Jeun SS (2014). Mesenchymal stem cells expressing brain-derived neurotrophic factor enhance endogenous neurogenesis in an ischemic stroke model. Biomed Res Int.

[33] Jeong HH, Piao S, Ha JN, Kim IG, Oh SH, Lee JH, Cho HJ, Hong SH, Kim SW, Lee JY (2013). Combined therapeutic effect of udenafil and adipose-derived stem cell (ADSC)/brain-derived neurotrophic factor (BDNF)-membrane system in a rat model of cavernous nerve injury. Urology.

[34] Kim JH, Choi SC, Park CY, Park JH, Choi JH, Joo HJ, Hong SJ, Lim DS (2016). Transplantation of immortalized CD34+and CD34-adipose-derived stem cells improve cardiac function and mitigate systemic pro-inflammatory responses. PLoS ONE.

[35] Kim JM, Lee ST, Chu K, Jung KH, Song EC, Kim SJ, Sinn DI, Kim JH, Park DK, Kang KM (2007). Systemic transplantation of human adipose stem cells attenuated cerebral inflammation and degeneration in a hemorrhagic stroke model. Brain Res.

[36] Kim S, Chang KA, Kim JA, Park HG, Ra JC, Kim HS, Suh YH (2012). The preventive and therapeutic effects of intravenous human adipose-derived stem cells in Alzheimer's disease mice. PLoS ONE.

[37] Kuhbier JW, Weyand B, Radtke C, Vogt PM, Kasper C, Reimers K (2010). Isolation, characterization, differentiation, and application of adipose-derived stem cells. Adv Biochem Eng Biot.

[38] Kuzma-Kozakiewicz M, Marchel A, Kaminska A, Gawel M, Sznajder J, Figiel-Dabrowska A, Nowak A, Maj E, Krzesniak NE, Noszczyk BH (2018). Intraspinal transplantation of the adipose tissue-derived regenerative cells in amyotrophic lateral sclerosis in accordance with the current experts' recommendations: choosing optimal monitoring tools. Stem Cells Int.

[39] Lee M, Ban JJ, Kim KY, Jeon GS, Im W, Sung JJ, Kim M (2016). Adipose-derived stem cell exosomes alleviate pathology of amyotrophic lateral sclerosis in vitro. Biochem Bioph Res Co.

[40] Lee M, Ban JJ, Yang S, Im W, Kim M (2018). The exosome of adipose-derived stem cells reduces beta-amyloid pathology and apoptosis of neuronal cells derived from the transgenic mouse model of Alzheimer's disease. Brain Res.

[41] Leu S, Lin YC, Yuen CM, Yen CH, Kao YH, Sun CK, Yip HK (2010). Adipose-derived mesenchymal stem cells markedly attenuate brain infarct size and improve neurological function in rats. J Transl Med.

[42] Li X, Zheng W, Bai HY, Wang J, Wei RL, Wen HT, Ning HB (2016). Intravenous administration of adipose tissue-derived stem cells enhances nerve healing and promotes BDNF expression via the TrkB signaling in a rat stroke model. Neuropsych Dis Treat.

[43] Liberale L, Gaul DS, Akhmedov A, Bonetti NR, Nageswaran V, Costantino S, Pahla J, Weber J, Fehr V, Vdovenko D (2020). Endothelial SIRT6 blunts stroke size and neurological deficit by preserving blood-brain barrier integrity: a translational study. Eur Heart J.

[44] Luo HL, Zhang YJ, Zhang ZQ, Jin Y (2012). The protection of MSCs from apoptosis in nerve regeneration by TGF beta 1 through reducing inflammation and promoting VEGF-dependent angiogenesis. Biomaterials.

[45] Lyden P, Shuaib A, Ng K, Levin K, Atkinson RP, Rajput A, Wechsler L, Ashwood T, Claesson L, Odergren T (2002). Clomethiazole acute stroke study in ischemic stroke (CLASS-I)—final results. Stroke.

[46] Morris GF, Bullock R, Marshall SB, Marmarou A, Maas A, Marshall LF, Investigators S (1999). Failure of the competitive N-methyl-D-aspartate antagonist Selfotel (CGS 19755) in the treatment of severe head injury: results of two Phase III clinical trials. J Neurosurg.

[47] Peng W, Gao TJ, Yang ZL, Zhang SC, Ren ML, Wang ZG, Zhang B (2012). Adipose-derived stem cells induced dendritic cells undergo tolerance and inhibit Th1 polarization. Cell Immunol.

[48] Qin YR, Zhou CK, Wang NH, Yang H, Gao WQ (2015). Conversion of adipose tissue-derived mesenchymal stem cells to neural stem cell-like cells by a single transcription factor, Sox2. Cell Reprogram.

[49] Radhakrishnan S, Trentz OA, Reddy MS, Rela M, Kandasamy M, Sellathamby S (2019). In vitro transdifferentiation of human adipose tissue-derived stem cells to neural lineage cells-a stage-specific incidence. Adipocyte.

[50] Reid AJ, Sun M, Wiberg M, Downes S, Terenghi G, Kingham PJ (2011). Nerve repair with adipose-derived stem cells protects dorsal root ganglia neurons from apoptosis. Neuroscience.

[51] Romanov YA, Darevskava AN, Merzlikina NV, Buravkova LB (2005). Mesenchymal stem cells from human bone marrow and adipose tissue: Isolation, characterization, and differentiation potentialities. B Exp Biol Med..

[52] Russell KA, Chow NHC, Dukoff D, Gibson TWG, LaMarre J, Bette DH, Koch TG (2016). Characterization and Immunomodulatory effects of canine adipose tissue- and bone marrow-derived mesenchymal stromal cells. PLoS ONE.

[53] Schmidt A, Diederich K, Strecker JK, Geng B, Hoppen M, Duning T, Schabitz WR, Minnerup J (2015). Progressive cognitive deficits in a mouse model of recurrent photothrombotic stroke. Stroke.

[54] Sherman DG, Bes A, Easton JD, Hacke W, Kaste M, Polmar SH, Zivin JA, Fieschi C, Miller P, Schoenfeld D (2001). Use of anti-ICAM-1 therapy in ischemic stroke—results of the Enlimomab Acute Stroke Trial. Neurology.

[55] Tobin MK, Bonds JA, Minshall RD, Pelligrino DA, Testai FD, Lazarov O (2014). Neurogenesis and inflammation after ischemic stroke: what is known and where we go from here. J Cerebr Blood F Met.

[56] Urrutia DN, Caviedes P, Mardones R, Minguell JPJ, Vega-Letter AM, Jofre CM (2019). Comparative study of the neural differentiation capacity of mesenchymal stromal cells from different tissue sources: an approach for their use in neural regeneration therapies. PLoS ONE.

[57] Wang J, Zhao HP, Fan ZB, Li GW, Ma QF, Tao Z, Wang RL, Feng J, Luo YM (2017). Long noncoding RNA H19 promotes neuroinflammation in ischemic stroke by driving histone deacetylase 1-dependent M1 microglial polarization. Stroke.

[58] Wang JH, Liu N, Du HW, Weng JS, Chen RH, Xiao YC, Zhang YX (2008). Effects of adipose-derived stem cell transplantation on the angiogenesis and the expression of bFGF and VEGF in the brain post focal cerebral ischemia in rats. Chin J Cell Mol Imm.

[59] Wang YL, Guo XL, Liu J, Zheng ZC, Liu Y, Gao WY, Xiao J, Liu YQ, Li Y, Tang ML (2020). Olfactory ensheathing cells in chronic ischemic stroke: a phase 2, double-blind, randomized, controlled trial. J Neurorestoratol.

[60] Wu CX, Lin JL, Hong M, Choudhury Y, Balani P, Leung D, Dang LH, Zhao Y, Zeng JM, Wang S (2009). Combinatorial control of suicide gene expression by tissue-specific promoter and microRNA regulation for cancer therapy. Mol Ther.

[61] Wu ZM, Zeng MY, Li C, Qiu HY, Feng HX, Xu XN, Zhang HY, Wu J (2019). Time-dependence of NIHSS in predicting functional outcome of patients with acute ischemic stroke treated with intravenous thrombolysis. Postgrad Med J.

[62] Xie J, Jones TJ, Feng DN, Cook TG, Jester AA, Yi R, Jawed YT, Babbey C, March KL, Murphy MP (2017). Human Adipose-derived stem cells suppress elastase-induced murine abdominal aortic inflammation and aneurysm expansion through paracrine factors. Cell Transpl.

[63] Xu J, Peng H, Kang N, Zhao Z, Lin JHC, Stanton PK, Kang J (2007). Glutamate-induced exocytosis of glutamate from astrocytes. J Biol Chem.

[64] Xu JY, Sastry BR (2005). Benzodiazepine involvement in LTP of the GABA-ergic IPSC in rat hippocampal CA1 neurons. Brain Res.

[65] Yan YF, Ma T, Gong K, Ao Q, Zhang XF, Gong YD (2014). Adipose-derived mesenchymal stem cell transplantation promotes adult neurogenesis in the brains of Alzheimer's disease mice. Neural Regen Res.

[66] Yeh DC, Chan TM, Harn HJ, Chiou TW, Chen HS, Lin ZS, Lin SZ (2015). Adipose tissue-derived stem cells in neural regenerative medicine. Cell Transpl.

[67] Zavan B, Michelotto L, Lancerotto L, Della Puppa A, D'Avella D, Abatangelo G, Vindigni V, Cortivo R (2010). Neural potential of a stem cell population in the adipose and cutaneous tissues. Neurol Res.

[68] Zhang HT, Liu ZL, Yao XQ, Yang ZJ, Xu RX (2012). Neural differentiation ability of mesenchymal stromal cells from bone marrow and adipose tissue: a comparative study. Cytotherapy.

[69] Zhang R, Zhong CK, Zhang YH, Xie XW, Zhu ZB, Wang AL, Chen CS, Peng YB, Peng H, Li QW (2019). Immediate antihypertensive treatment for patients with acute ischemic stroke with or without history of hypertension a secondary analysis of the CATIS randomized clinical trial. Jama Netw Open.

[70] Zhang Y, Deng H, Hu Y, Pan C, Wu GF, Li Q, Tang ZP (2019). Adipose-derived mesenchymal stem cells stereotactic transplantation alleviate brain edema from intracerebral hemorrhage. J Cell Biochem.

[71] Zhang Y, Tang WY, Su XW, Dong BX, Wang Q, Wang ZY, Yang YX, Qu SQ, Luan Z (2018). Immunological effects of the intraparenchymal administration of allogeneic and autologous adipose-derived mesenchymal stem cells after the acute phase of middle cerebral artery occlusion in rats. J Transl Med.

[72] Zhou F, Gao SN, Wang L, Sun CX, Chen L, Yuan P, Zhao HY, Yi Y, Qin Y, Dong ZQ (2015). Human adipose-derived stem cells partially rescue the stroke syndromes by promoting spatial learning and memory in mouse middle cerebral artery occlusion model. Stem Cell Res Ther.

[73] Zuk PA (2010). The adipose-derived stem cell: looking back and looking ahead. Mol Biol Cell.

