# Supplementary material for: Rebuilding hippocampus neural circuit with hADSC-derived neuron cells for treating ischemic stroke
Source: Cell Biosci. 2022 Apr 4;12:40. doi: 10.1186/s13578-022-00774-x (PMC8981707; doi:10.1186/s13578-022-00774-x)
Supplement: Supplementary file 1 — Additional file 1. Additional figures. [file 13578_2022_774_MOESM1_ESM.pdf]

Fig. Suppl. 1 Evaluating hADSC-NCs in terms of health, migration and human origin in the hippocampus.

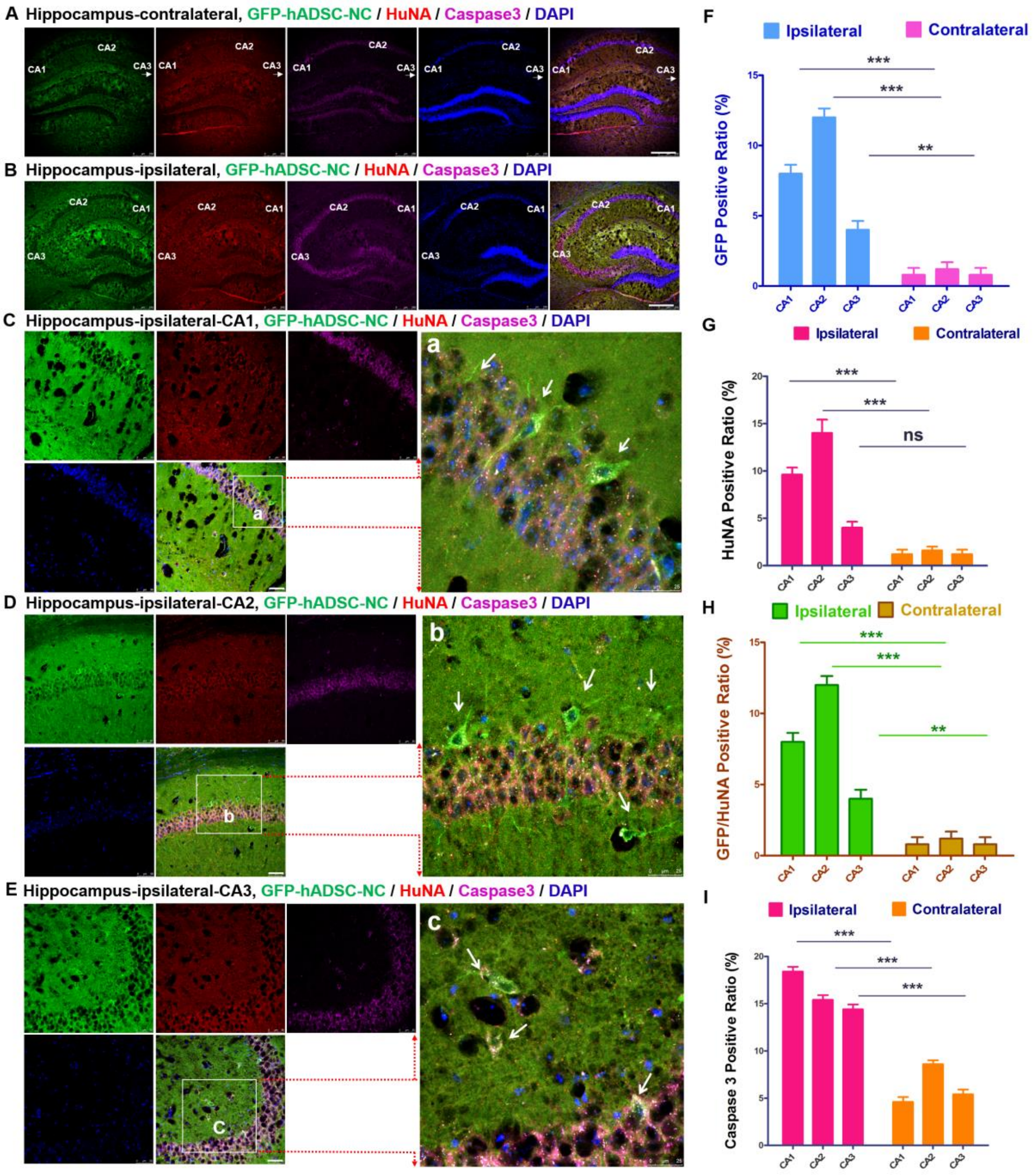

Fig. Suppl.1 Evaluating hADSC-NCs in terms of health, migration and human origin in the hippocampus. A shows the overview of the immunohistochemical staining of the hippocampus on the contralateral side with human nuclear antigen antibody (HuNA) and Caspase 3, B shows the

ipsilateral side. C shows hADSC-NCs integrated into the ipsilateral hippocampus CA1 area and their expression of HuNA and Caspase 3. D shows hADSC-NC integrated into the ipsilateral hippocampus CA2 area, and their expression of HuNA and Caspase 3. E shows hADSC-NC integrated into the ipsilateral hippocampus CA3 area and their expression of HuNA and Caspase 3. a, b, c shows the zoomed-in view of cells in C, D, and E. F-I shows the statistical analysis of the GFP^{+} (hADSC-NC), HuNA^{+} and Caspase 3^{+} percentages in each area of the hippocampus. n=5, Scale bar=250 μm in A-B, scale bar=50 μm in C-E. * indicates P<0.05, ** indicates P<0.001. *** indicates P<0.0005.

Fig. Suppl. 2 Determination of the proliferative properties of transplanted hADSC-NCs *in vivo*.

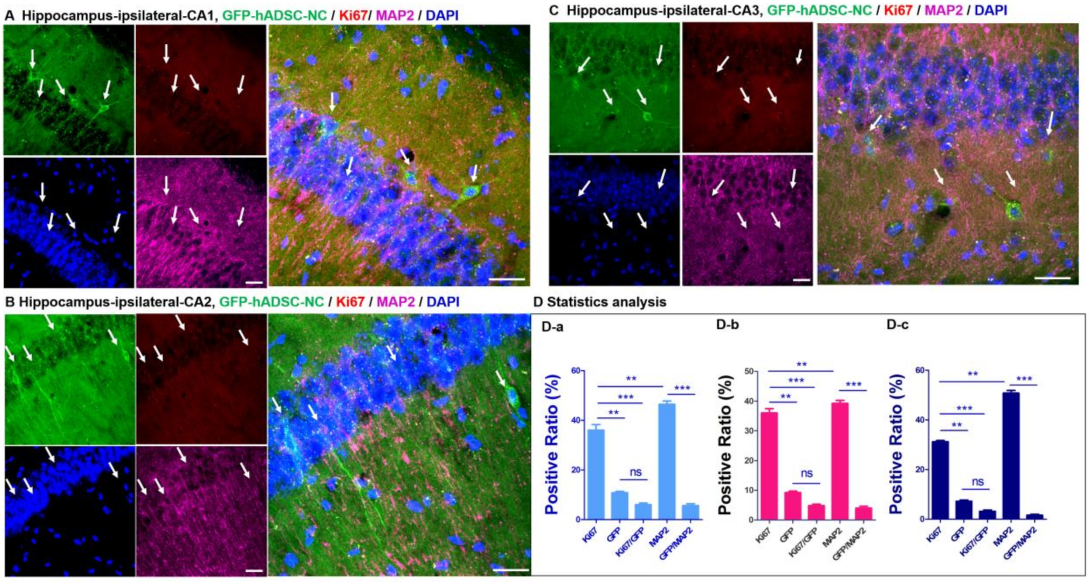

Fig. Suppl.2 Determination of the proliferative properties of transplanted hADSC-NCs *in vivo*. GFP-positive hADSC-NCs were Ki67-positive and MAP2-positive in the CA1 (A), CA2 (B) and CA3 (C) regions. Statistical analysis of the cell positive percentages is shown in D, D-a for the CA1 area, D-b for the CA2 area and D-c for the CA3 area. n=5, Scale bar=25 μm. * indicates P<0.05, ** indicates P<0.001. *** indicates P<0.0005.

Fig. Suppl.3 hADSC-NCs modulate the MCAO mouse immune system locally rather than systemically in serum.

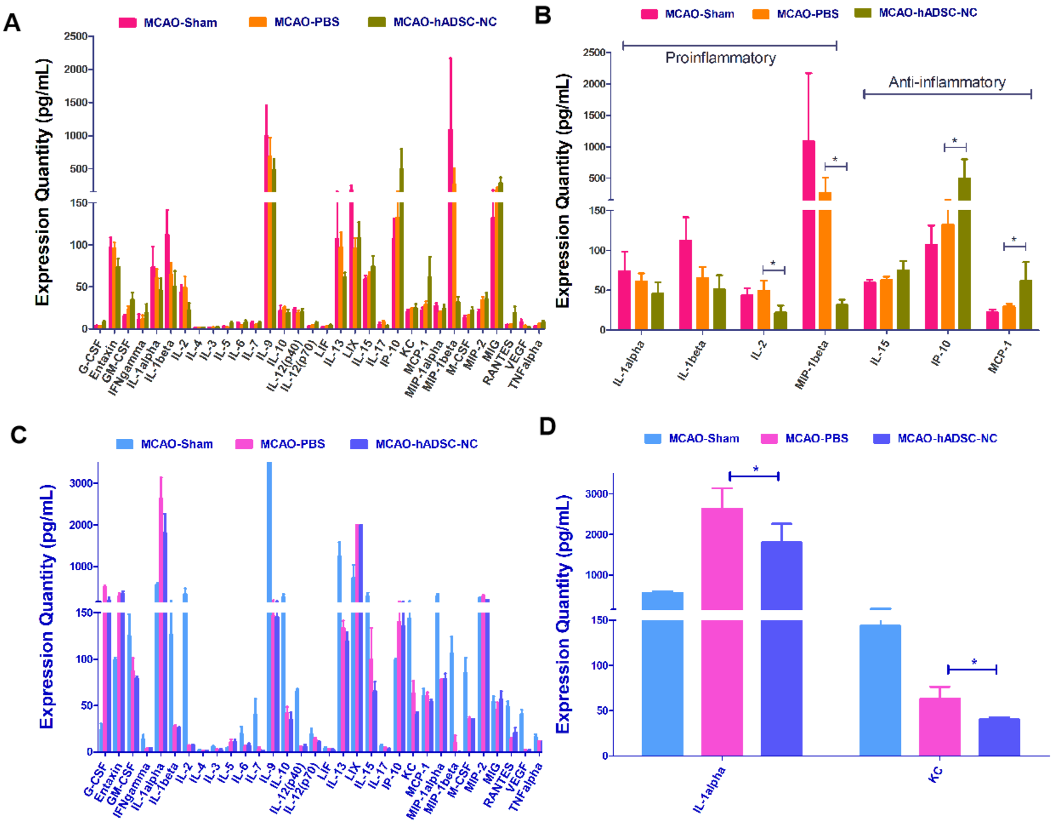

Fig. Suppl.3 hADSC-NCs modulate the MCAO mouse immune system locally rather than systemically in serum.A shows the whole immune factor profile in local brain tissue. B shows the significant variations of factors in brain tissue. C shows the immune factor profile in serum. D shows the significant variations of factors in serum.
